# Urea-mediated dissociation alleviate the false-positive *Treponema pallidum*-specific antibodies detected by ELISA

**DOI:** 10.1371/journal.pone.0212893

**Published:** 2019-03-05

**Authors:** Qiang Wang, Yan Lei, Xiaolan Lu, Guangrong Wang, Qin Du, Xiaolan Guo, Yan Xing, Guoyuan Zhang, Dongsheng Wang

**Affiliations:** 1 Department of Laboratory Medicine, Affiliated Hospital of North Sichuan Medical College, Nanchong, Sichuan, P.R. China; 2 Faculty of Laboratory Medicine, Center for Translational Medicine, North Sichuan Medical college, Nanchong, Sichuan, P.R. China; Consiglio Nazionale delle Ricerche, ITALY

## Abstract

The serological detection of antibodies to *Treponema pallidum* is essential to the diagnosis of syphilis. However, for the presence of cross-reaction, the specific antibody tests [e.g., enzyme-linked immunosorbent assay (ELISA)] always have false-positive results. In this study, we derived and validated the dissociation of urea in an attempt to alleviate the situation of false-positive antibodies to *T*. *pallidum* detected by ELISA. Six serum samples that were false-positive antibodies to *T*. *pallidum* detected by ELISA, and 16 control serum samples (8 sera positive for both specific IgG and IgM, and 8 IgG-positive and IgM-negative sera) were collected to select the appropriate dissociated concentration and time of urea. Our goal was to establish improved an ELISA method based on the original detection system of ELISA. The sensitivity of the improved ELISA was evaluated by 275 serum samples with class IgM-positive antibodies to *T*. *pallidum*. At 6 mol/L with 10 minutes dissociation of urea, 6 samples with false-positive antibodies to *T*. *pallidum* were converted to negative, and compared with true-positive antibodies to *T*. *pallidum*. The sensitivity of the improved ELISA was 100% by detecting the class IgM-positive antibodies to *T*. *pallidum* in sera of patients with syphilis. Considering the importance at the diagnosis of syphilis, antibodies to *T*. *pallidum* in serum samples should be retested by the improved ELISA method to avoid false-positive results.

## Introduction

Syphilis is a sexually transmitted disease caused by the spirochete *Treponema pallidum* (subsp. Pallidum) and is characterised by widespread tissue dissemination and chronic infection[1–2]. The disease continues to be a significant public health problem worldwide. The World Health Organization (WHO) estimates that 12 million new cases of syphilis occur each year, and more than 90% of them are in developing countries[3]. In 2012, WHO estimated 350,000 cases of adverse pregnancy outcomes due to syphilis, and congenital syphilis remains a leading cause of stillbirths and death among neonates in many developing countries[3–4]. In China, the total rate of cases of syphilis was approximately 0.2 cases per 100,000 persons in 1993; however, primary and secondary syphilis increased to 5.7 to 10.0 cases per 100,000 persons in 2005. Congenital syphilis also increased greatly, from approximately 0.01 cases per 100,000 livebirths in 1991 to 19.8 to 25.0 cases per 100,000 livebirths in 2005[5–6].

Because of wide clinical manifestations, the diagnosis of syphilis is difficult[7]. According to the US Centers for Disease Control and Prevention (CDC) and the International Union against Sexually Transmitted Infections (IUSTI) guidelines for the diagnosis and management of patients with syphilis, the serological detection of *T*. *pallidum*-specific antibodies is of particular importance[8–9]. Serological detections are divided into non-treponemal and treponemal detections. The most widely used non-treponemal detections are Venereal Disease Research Laboratory (VDRL) and Rapid Plasma Reagin (RPR). Because of low specificity and sensitivity, non-treponemal detections can be used merely to assess the activity of infection and monitor therapy[10–13]. Treponemal detections are the other type of diagnostic detections, and include the Treponema Pallidum Particle Agglutination (TPPA) test, the Fluorescent Treponemal Antibody Absorption (FTA-ABS) test, the Treponema Pallidum Haemagglutination (TPHA) test, chemiluminescent immunoassay (CLIA), and enzyme-linked immunoassay (ELISA)[8,14–17]. Compared with the other methods, ELISA is widely used in the diagnosis of syphilis in China due to its low cost and easy operation, and the specificity of this method is approximately 98%, indicating that there is a certain amount of false-positive results. This situation may cause confusion among the examinees and clinical practitioners, and further to professional institutions for more expensive confirmation experiments, such as Western blott [18–22]. TpN15, TpN17, and TpN47 are three specific lipoproteins that are present in the membrane surface of *T*. *pallidum*, and also are the three major immunogens in experimental and natural *T*. *pallidum* infection[23–24]. During *T*. *pallidum* infection, the earliest produced antibody is against TpN47, followed by TpN15 and TpN17[23,25]. The detection principle of TP-ELISA kits often uses a "antigen" sandwich complex, the recombinant composite antigens of TpN15, TpN17, and TpN47 to combine with the plate to improve the sensitivity, and can simultaneously detect IgM and IgG type antibodies[26]. However, this configuration may also increase the opportunity of cross-reaction between specific antigen and cross-antibody, and leads to false-positive treponemal detections.

The affinity of cross-reaction between a specific antigen and a cross-antibody is lower than the specific reaction[27]. Urea is a dissociating agent that can be used to assess the avidity of IgG, such as Toxo-plasma IgG, in different detecting systems[28–29]. We therefore investigated whether the dissociation of urea could be used to distinguish true-positive from false-positive results of antibodies to *T*. *pallidum* detected by ELISA, and whether the configuring mode of the improved ELISA, combined with the dissociation of urea, could alleviate the situation of false-positive antibodies to *T*. *pallidum* detected by ELISA. If so, this study can provide an useful solution for all clinical laboratories who have false-positive antibodies to *T*. *pallidum*, even other pathogen antibodies detected by ELISA.

## Materials and methods

### Study subjects

A total of 297 sera, obtained from the Affiliated Hospital of North Sichuan Medical College Hospital (Sichuan China) from July 2013 to October 2016, were used in the current study. Of the 297 sera, 6 sera that were false-positive antibodies to *T*. *pallidum* detected by ELISA were obtained from patients with no syphilis clinical symptoms and a negative epidemiological history, and 16 control sera (8 sera positive for both specific IgG and IgM, 8 IgG-positive and IgM-negative sera) were collected in this study to select the best dissociated concentration and time of urea. The remaining 275 sera (IgM positive), obtained from patients with *T*. *pallidum* primary infection, were collected to evaluate the performance of the method of improved ELISA, which was based on the original detection system of ELISA.

**Fig 1:**
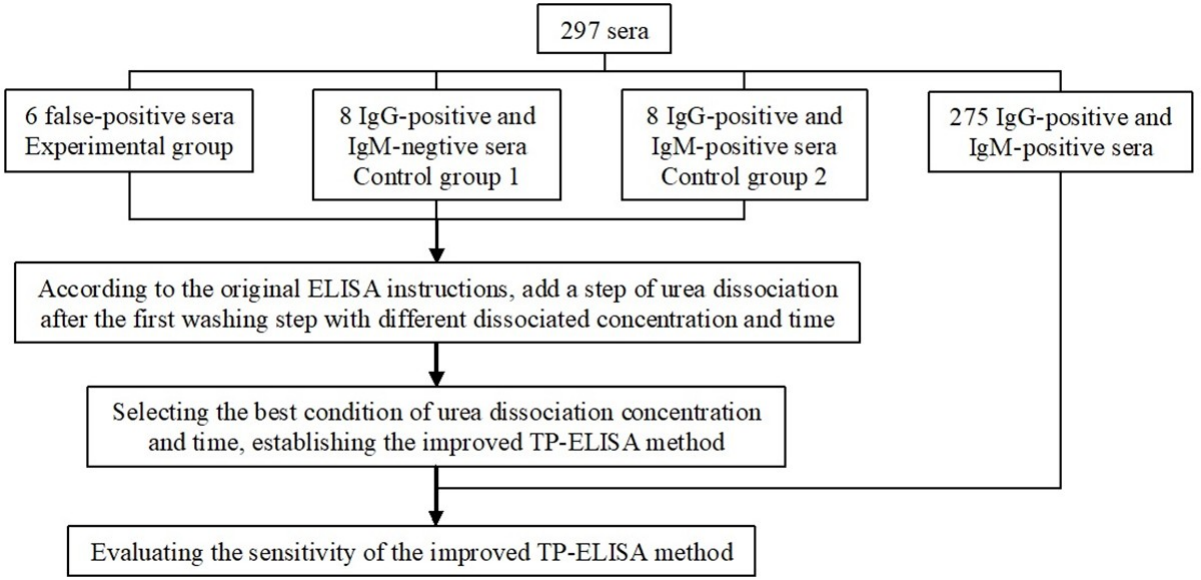
Flowchart depicting study procedures.

All the 297 serum samples used in this study had been stored frozen at -80°C after being detected by ELISA for the class IgG and/or IgM antibodies to *T*. *pallidum* (Anti-*T*. *pallidum* antibody ELISA test kit, WanTai Biopharm, China), ELISA for the class IgM antibodies to *T*. *pallidum* (Anti-*T*. *pallidum* antibody(IgM) ELISA test kit, EUROIMMUN, Germany), and CLIA for quantifying the class IgG and/or IgM antibodies to *T*. *pallidum* (ARCHITECT Syphilis TP, Abbott Japan Co., Japan). All serums were thawed at room temperature and then centrifuged at 2,583×g for subsequent tests. The study procedures has been depicted in the form of a flowchart in Fig 1.

### Western blot analysis

The western blot test kit (EUROIMMUN, Germany) provides a qualitative in vitro assay for the class IgG antibodies against *T*. *pallidum* in serum or plasma for the diagnosis of infections with *T*. *pallidum* and associated diseases (Lues). Six false-positive samples and 16 true-positive control samples were detected by western blot analysis to ensure the reliability of the follow-up experiment results. The kit contains test strips with electrophoretically separated antigens of *T*. *pallidum*. The blot strips were blocked and incubated in the first reaction step with diluted patient samples. In the case of positive samples, specific antibodies of the class IgG antibodies bound to the antigens. To detect the bound antibodies, a second incubation was performed using an enzyme-labelled anti-human IgG (alkaline-phosphatase conjugate) catalysing a colour reaction. The results were interpreted as follows: no bands of specific antigens, negative; one distinctive band of specific antigen (Tp15 kDa, Tp17 kDa, Tp45 kDa or Tp47 kDa), borderline; more than one distinctive band of the specific antigens, positive.

### Urea dissociation test

Six false-positive samples and 16 control samples were collected to select the appropriate dissociated concentration and dissociated time of urea. The steps were as follows: first, 100 μL of sera were added to the wells of the plates coated with *T*. *pallidum*-specific antigens(TpN15, TpN17, and TpN47), and incubated at 37°C for 60 minutes. After the first washing, 100 μL of phosphate-buffered saline (PBS) solution (containing 0 mol/l, 2 mol/L, 4 mol/L, 6 mol/L, 8 mol/L, and 10 mol/L urea in different wells) was added and incubated at 37°C for 5 minutes, 10 minutes, and 20 minutes for different plates. After the second wash, *T*. *pallidum*-specific antigens labelled by horseradish peroxidase were added into the reaction system to form an immune sandwich complex. After the third wash to remove unbound substances, we added substrate for the colour reaction. The results were interpreted by the ratios of the sample optical density value and the cut-off optical density value (S/CO), as follows: S/CO equal to 1.00 or greater, positive; S/CO less than 1.00, negative. We followed the original ELISA cut-off optical density value criterion, which was 0.18Abc plus negative control optical density value when its^’^ value was more than 0.05Abc, if less than 0.05Abc, calculated at 0.05Abc.

### Sensitivity test

The sensitivity test was followed by the urea dissociation test for confirming the appropriate dissociated concentration and time. A total of 275 samples, all with IgM-positive antibodies to *T*. *pallidum*, were used to evaluate the sensitivity of the improved ELISA method, and the assay procedure confirmed the concentration and time.

## Results

### Laboratory features of study subjects

**Fig 2:**
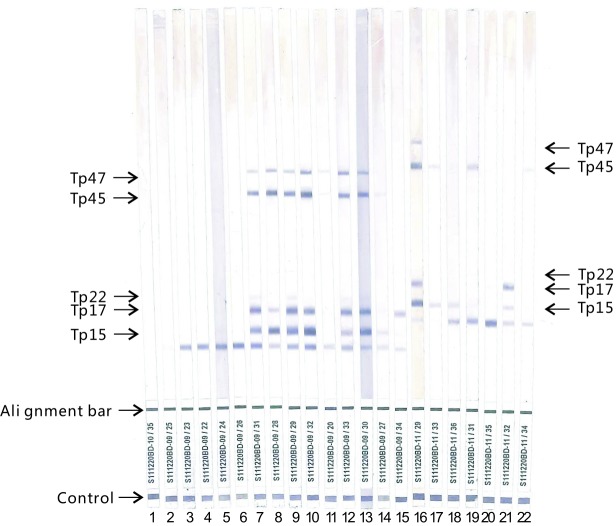
Western blot analysis of human sera for urea dissociation test. The positions of the four diagnostic T. pallidum antigens(Tp15 kDa, Tp17 kDa, Tp45 kDa and Tp47 kDa) were indicated on the left and right for different lot number of the Western blot test kit[S111220BD-09(lane2-15), S111220BD-10(lane1) and S111220BD-11(lane16-22)]. Lane 1–6: sera obtained from patients with false positive ELISA results, no bands of specific antigens of T. pallidum. Lane 7–14: sera obtained from patients with positive ELISA and CLIA results, more than one distinctive band of the specific antigens. Lane 15–22: sera obtained from patients with positive ELISA, ELISA-IgM and CLIA results, one distinctive band of the specific antigens at least.

We collected the serum samples required for this study with targets. Six false-positive samples were detected positive for the class IgG and/or IgM antibodies to *T*. *pallidum* by ELISA, but negative for the class IgG and/or IgM antibodies to *T*. *pallidum* by CLIA and for the class IgG antibodies to *T*. *pallidum* by western blot assay. Sixteen control samples tested positive by ELISA and CLIA, and positive or borderline by western blot assay (Fig 2). The class IgM antibodies to *T*. *pallidum* were detected, of which 8 samples were positive and 8 samples were negative. In addition, the remaining 275 samples were all positive for the class IgG and/or IgM antibodies to *T*. *pallidum* by ELISA test, were all low reactive tested by CLIA, and were all positive for the class IgM antibodies to *T*. *pallidum* by ELISA. All the results are shown in Table 1.

**Table 1:** The results of antibodies to *T*. *pallidum* of 297 serum samples detected by different methods.

Study subjects	ELISA	ELISA for IgM	CLIA(S/CO)	Western blot
6 false-positive sera	P	N	<1.00	N
16 control sera				
8 IgG-positive and IgM-negative sera	P	N	3.57±0.60	P
8 IgG-positive and IgM-positive sera	P	P	3.28±0.42	B or P
275 sera	P	P	3.23±0.71	—

### Selection of dissociated concentration and time of urea

**Fig 3:**
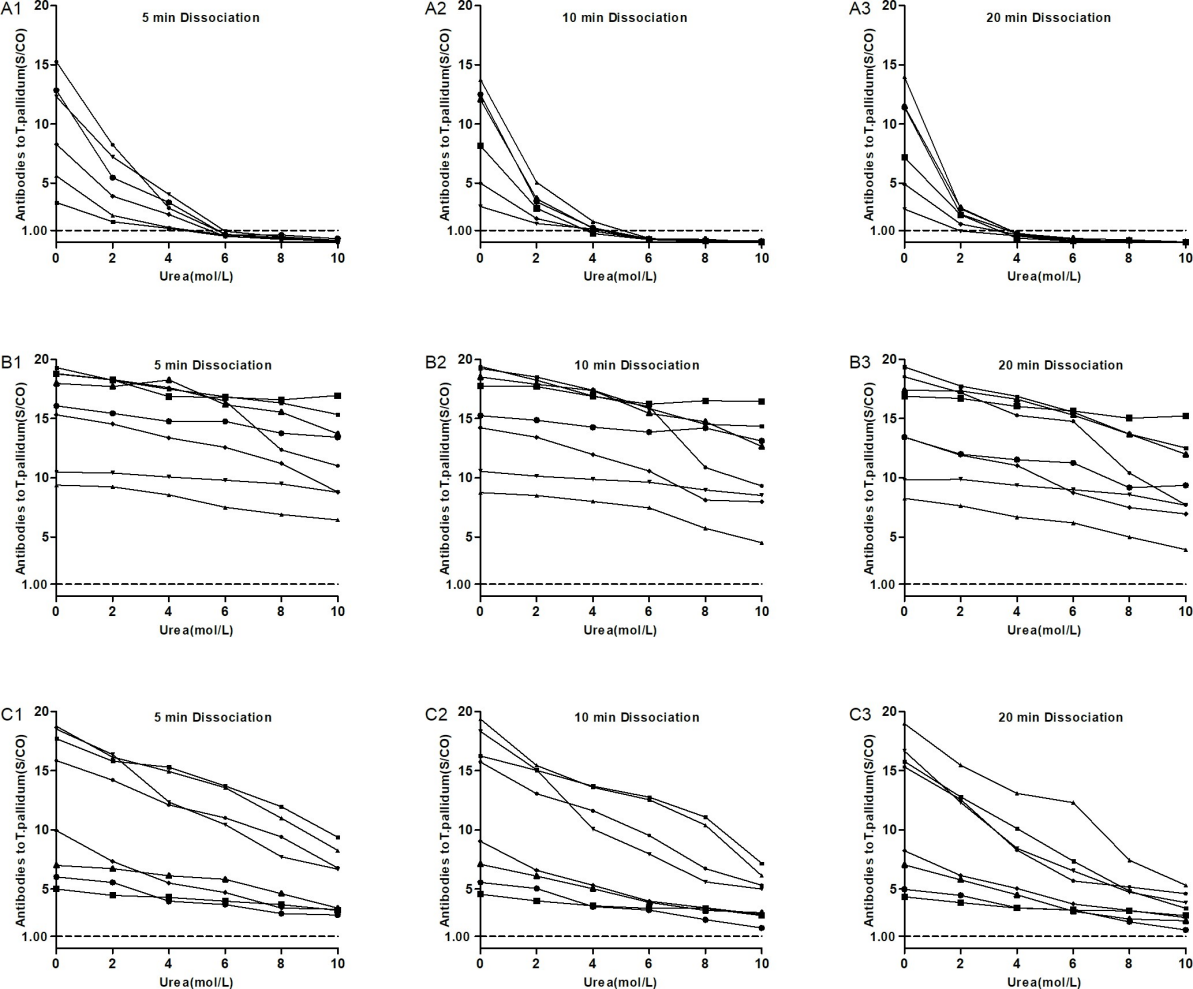
Urea dissociation test to determine appropriate dissociation concentration and time. The curve showed continuous changes of dissociation results at 0mol/L, 2mol/L, 4mol/L, 6mol/L, 8mol/L and 10mol/L dissociation concentration with 5min, 10min and 20min dissociation time of urea for different group. A1~A3: for 6 false-positive samples. B1~B3: for 8 IgG-positive and IgM-negative samples as control group. C1~C3: for 8 IgG-positive and IgM-positive samples as another control group.

The value of S/CO of antibodies to *T*. *pallidum* of 6 false-positive samples decreased with the increase of dissociated concentration and time (Fig 3A1 to 3A3), and the reduced range was higher than that of the 16 control samples (Fig 3B1 to 3C3). Of the 16 control samples, the value of S/CO of antibodies to *T*. *pallidum* of 8 samples with IgM positivity were reduced more than that of samples with IgM negativity. When the dissociated concentration and time of urea were selected at 6 mol/L and 10 minutes, respectively, as detecting conditions of improved ELISA, all the values of S/CO of antibodies to *T*. *pallidum* of the 6 false-positive samples were below 1.00, compared with 16 control samples above 1.00, and achieved a best distinction between true-positive and false-positive samples.

### Sensitivity of improved ELISA

**Fig 4:**
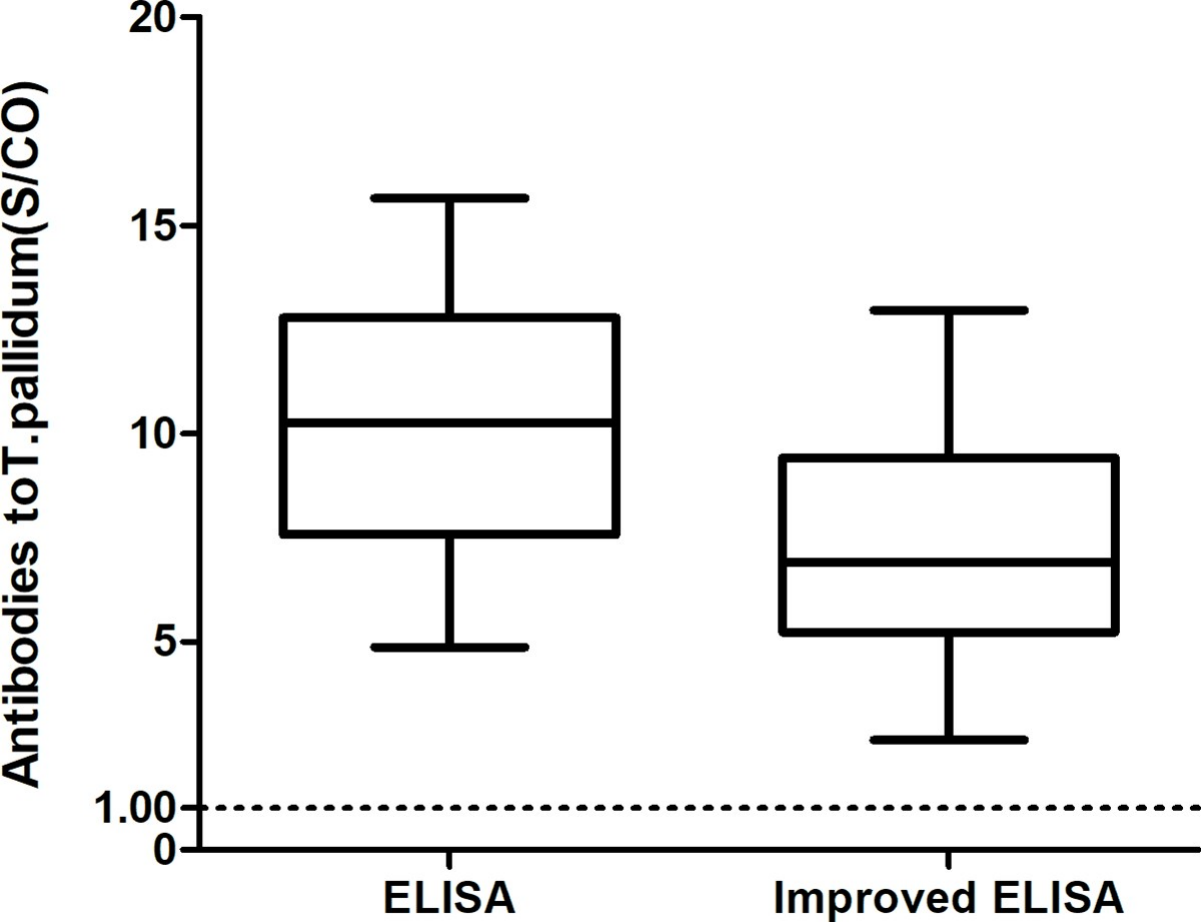
Comparation the detected results of antibodies to T.pallidum between ELISA and improved ELISA. The value of S/CO of antibodies to T.pallidum detected by improved ELISA were all reduced, compared to ELISA, but all greater than 1.00. The operation steps of ELISA were strictly followed by kit instruction, and improved ELISA was inserted one step as adding PBS contained 6mol/L for 10 minutes after the first washing step.

Compared with ELISA, the value of S/CO of antibodies to *T*. *pallidum* of 275 samples detected by improved ELISA were all reduced, but all greater than 1.00. No false-negative results were found, indicating that the sensitivity of the improved ELISA method to detect antibodies to *T*. *pallidum* was 100% (Fig 4).

## Discussion

China has an extensive number of people with syphilis infection, and the demand for the detection of antibodies to *T*. *pallidum* is also very large[5–6]. Because of the advantages of simple operation, simple equipment, and low cost, ELISA is a classic method for detecting antibodies to *T*. *pallidum*, and is adopted by most clinical laboratories. It has been reported that the specificity of ELISA is approximately 98.00%, which means some false-positive cases exist[21–22]. Therefore, developing an improved method based on the original detection system of ELISA is very practical as the confirmatory test for antibodies to *T*. *pallidum*, after being detected positive by ELISA.

In this study, we used the function of urea dissociation and relied on the original detection system of ELISA to deal with the situation of false-positivity of antibodies to *T*. *pallidum* detected by ELISA. The principle of selecting the dissociation concentration and dissociation time of the urea was as follows: keeping the S/CO value of antibodies to *T*. *pallidum* of false-positive samples away from 1.00 as far as possible, while minimising the S/CO reduction in positive samples of antibodies to *T*. *pallidum*. The S/CO results of 6 false-positive samples of antibodies to *T*. *pallidum* were all lower than 1.00 when the dissociation time of 5 minutes and the dissociation concentration of 6 mol/L, but one S/CO result of antibodies to *T*. *pallidum* was 0.94 (Fig 3A1). This condition may cause a false-positive result in the subsequent application, leading to the misinterpretation of the results. When the dissociation concentration was 6 mol/L and the dissociation time was 20 minutes, the selection principle was achieved, but the detection performance was not improved, and extended detection time, compared with 6 mol/L and 10 minutes condition (Fig 3A3, 3B3 and 3C3). Therefore, the dissociation time of 10 minutes and the dissociation concentration of 6 mol/L should be the best condition, which could properly achieve distinguishing true-positive and false-positive results (Fig 3A2, 3B2 and 3C2).

As compared with the class IgG antibodies, the class IgM antibodies have a lower affinity with the antigen, which is also verified by the experimental results[30]. Therefore, in the selection of control sera, the sera with low reactivity in CLIA detection results were selected, which included 8 samples with positive class IgM antibodies to *T*. *pallidum*, whereas the samples for sensitivity assessment were the samples whose reactivity was low in CLIA detection and whose IgM antibodies to *T*. *pallidum* were positive. As compared with ELISA, there was a significant decrease in the S/CO results when the improved ELISA was used to detect the positive samples of the class IgM antibodies to *T*. *pallidum*. However, under the conditions of the selected dissociation concentration and dissociation time, the S/CO results of all the positive samples of the class IgM antibodies to *T*. *pallidum* were greater than 1.00, and the sensitivity of 275 samples was 100%, indicating that the improved ELISA basically would not cause the missing detection of positive specimens.

In this study, the principle and procedure of the improved ELISA and the original ELISA were almost identical, except the improved ELISA was inserted one step as adding PBS contained 6mol/L urea for 10 minutes after the first washing step. We did not evaluate the other performance of the improved ELISA, nor did we reset the critical value, but used the critical value judgment method of the original ELISA. Based on the following considerations: using the original ELISA reaction system can make the detection process simpler. Above all, this mode is suitable for almost all TP-ELISA reaction systems, and may be better applied and popularized.

Because of the difficulties in collecting ELISA false-positive samples, only 6 cases were collected in the laboratory within 3 years; thus, no definitive assessment was given to the specificity of improved ELISA in this study. However, it can be learned from the dissociation experiment that the experimental conditions resulting from the screening results basically would not cause a false-positive result.

In conclusion, in view of the importance of syphilis antibody detection in the syphilis diagnosis and the universality of ELISA in clinical laboratories, it is of high practical value to use the improved ELISA to confirm the preliminary screening positive samples of antibodies to *T*. *pallidum* obtained by ELISA. However, the syphilis diagnosis shall always be comprehensively assessed by a combination of laboratory serological detection results with the clinical symptoms and epidemiological survey in order to avoid the misdiagnosis due to cross-reaction as well as the missed diagnosis due to early syphilis infection and low IgM serum concentration, as Fig 3C1–3C3 and Fig 4 show.

## Supporting information

S1 Dataset The results of urea dissociation test and sensitivity test. (XLS)
